# Combined treatment with epigenetic agents enhances anti-tumor activity of MAGE-D4 peptide-specific T cells by upregulating the MAGE-D4 expression in glioma

**DOI:** 10.3389/fonc.2022.873639

**Published:** 2022-08-03

**Authors:** Shui-Qing Bi, Qing-Mei Zhang, Xia Zeng, Chang Liu, Wei-Xia Nong, Huan Xie, Feng Li, Li-Na Lin, Bin Luo, Ying-Ying Ge, Xiao-Xun Xie

**Affiliations:** ^1^ Department of Histology and Embryology, School of Preclinical Medicine, Guangxi Medical University, Nanning, Guangxi, China; ^2^ Department of Neurosurgery, The People’s Hospital of Guangxi Zhuang Autonomous Region, Guangxi Academy of Medical Sciences, Nanning, China; ^3^ Key laboratory of Preclinical Medicine, Education Department of Guangxi Zhuang Autonomous region, Nanning, China; ^4^ Department of Neurosurgery, The First Affiliated Hospital of Guangxi Medical University, Nanning, Guangxi, China

**Keywords:** decitabine, valproic acid, trichostatin A, MAGE-D4, cytotoxic T lymphocyte, glioma

## Abstract

**Objective:**

The study evaluated the efficacy of combined epigenetic drugs of decitabine (DAC), valproic acid (VPA), and trichostatin A (TSA) on immunotherapy against glioma.

**Methods:**

The expression and prognosis of MAGE-D4 in glioma were analyzed online, and the expression of MAGE-D4 and HLA-A2 in glioma induced by epigenetic drugs was detected by qRT-PCR, Western blot, and flow cytometry. The methylation status of the MAGE-D4 promoter was determined by pyrosequencing. An HLA-A2 restricted MAGE-D4 peptide was predicted and synthesized. An affinity assay and a peptide/HLA complex stability assay were performed to determine the affinity between peptide and HLA. CCK8 assay, CFSE assay, ELISA and ELISPOT were performed to detect the function of MAGE-D4 peptide-specific T cells. Flow cytometry, ELISA, and cytotoxicity assays were used to detect the cytotoxicity effect of MAGE-D4 peptide-specific T cells combined with epigenetic drugs against glioma *in vitro*. Finally, the glioma-loaded mouse model was applied to test the inhibitory effect of specific T cells on gliomas *in vivo*.

**Results:**

MAGE-D4 was highly expressed in glioma and correlated with poor prognosis. Glioma cells could be induced to express MAGE-D4 and HLA-A2 by epigenetic drugs. MAGE-D4-associated peptides were found that induce DCs to stimulate the highest T-cell activities of proliferation, IL-2 excretion, and IFN-γ secretion. MAGE-D4 peptide-specific T cells treated with TSA only or combining TSA and DAC had the most cytotoxicity effect, and its cytotoxicity effect on glioma cells decreased significantly after HLA blocking. *In vivo* experiments also confirmed that MAGE-D4-specific T cells inhibit TSA-treated glioma.

**Conclusion:**

MAGE-D4 is highly expressed in glioma and correlated with the prognosis of glioma. The novel MAGE-D4 peptide identified was capable of inducing MAGE-D4-specific T cells that can effectively inhibit glioma growth, and the epigenetic drug application can enhance this inhibition.

## Introduction

Brain cancer is considered the leading cause of cancer death, while its death rates are also increasing (1). However, gliomas account for more than 60% of all adult primary intracranial tumors (2). Most patients with malignant glioma survive for less than one year, even with surgery following radiation and chemotherapy (3–5). Therefore, novel therapeutic methods should be actively explored to improve the efficacy and prognosis of this fatal disease. In recent years, immunotherapy has attracted more and more attention due to its low toxicity and strong specificity and has become an ideal treatment method to improve the prognosis of patients with glioma. However, the key to immunotherapy is to find the target antigen with tumor characteristics and to make this therapy safe and effective.

Cancer Testis Antigen (CTA) is a kind of tumor-specific antigen that can be low expressed in normal tissues except testicular tissues and highly expressed in a range of tumor tissues. Melanoma associated antigen (MAGE) is a family of CTAs. Many studies are focused on MAGE in various tumors (6, 7). As a member of the MAGE family, MAGE-D4 expression is low in normal tissues and high in various tumors, including glioma (8–14). Therefore, MAGE-D4 may serve as a tumor immunotherapy target just like other members of the MAGE family, such as MAGE-A3 (15). However, T-cell immunotherapy alone is often ineffective. One part of the reason may result from antigen expression, for instance, heterogeneous expression of tumor antigen and low expression of HLA. To overcome this obstacle, a combination of epigenetic drugs with immunotherapy may be a better choice (16). There is a great amount of evidence that epigenetic modifications can regulate gene expression at the gene level. Methylation and histone acetylation are two types of epigenetic modification. Studies have shown that the use of demethylation agents (such as Decitabine and DAC) and/or histone acetylase inhibitors (such as Trichostatin A (TSA) and Valproic acid (VPA)) can increase the expression of CTA in tumors and enhance the immunotherapeutic antitumor effect (17–20). Our previous experiments also showed that demethylation by epigenetic drugs can increase the expression of MAGE-D4 in glioma (8, 13). Therefore, we consider that it is a possibility to apply epigenetic drugs as adjuvants for glioma immunotherapy.

In this study, with peptide binding prediction programs, we predicted HLA-A2 restricted peptides from MAGE-D4 because HLA-A*0201 is the most widely expressed HLA-I molecule in the Chinese population (21) and these candidate peptides were further confirmed by the binding affinity and stability of T2 cells to HLA-A*0201. Finally, anti-glioma immunity of T cells induced by MAGE-D4 peptides and epigenetic drugs was then investigated *in vitro* and *in vivo*. Our study may provide the rationale for the potential utility of the MAGE-D4 peptide in glioma immunotherapy.

## Materials and methods

### Online gene-expression analysis

Data from Gene Expression Omnibus (GEO, https://www.ncbi.nlm.nih.gov/geo/) and LinkedOmics (http://www.linkedomics.org) (22) were used to explore MAGE-D4 mRNA expression and survival analysis in human glioma. GEO data was from the GSE2223 dataset (23) containing 49 glioma tissues and 4 normal brain tissues as controls, whereas data from LinkedOmics contained 625 glioma samples.

### Cell lines, drugs and cytokines

The human glioma cell lines (U87-MG and U251) were obtained from the Cell Bank of Type Culture Collection of the Chinese Academy of Science (Shanghai, China). Decitabine (DAC, CAS No. A3656), valproic acid (VPA, CAS No. 99-66-1), and trichostatin A (TSA, CAS No. 58880-19-6) were purchased from Sigma-Aldrich Corporation (St. Louis, MO, USA). DAC and TSA were dissolved in DMSO to a concentration of 10 mM. Aliquots were stored at −80°C and diluted with media before being used. Drug treatments were divided into seven groups: (1) DAC group: cells were cultured in medium on the presence of 1 μM DAC for 120 h; (2) VPA group: cells were grown in medium containing 1 mM VPA for 120 h; (3) TSA group: cells were cultured in medium supplemented with 1 μM TSA for 24 h; (4) D + V group: cells were cultured with 1 μM DAC and 1 mM VPA for 120 h; (5) D + T group: cells were cultured with 1 μM DAC for 120 h, followed by 1 μM TSA for 24 h; (6) Control group: cells were cultured with 0.09% DMSO, which is the highest concentration of DMSO used in this study in consideration of the potential toxicity of DMSO to living cells. Human recombinant interleukin (IL) IL-2, IL-4, and human recombinant granulocyte/macrophage colony-stimulating factor (GM-CSF) were purchased from R&D Systems.

### Prediction and synthesis of peptides

Nine HLA-A2 restricted amino acid long peptides derived from the MAGE-D4 N-terminal protein with strong binding motifs for HLA-A2 were predicted using four well-established HLA peptide binding prediction programs: IEDB (https://www.iedb.org/) (24), netMHC (25) (http://www.cbs.dtu.dk/services/NetMHC/), EPIJEN (http://www.jenner.ac.uk/EpiJen) (26) and SYFPEITHI (http://www.syfpeithi.de/) (27). The HLA-A2 restricted peptide GILGFVFTL was used as a negative control (28). Peptides were produced commercially by Peptide Technologies of Wuhan Dangang Biotechnology Co., Ltd. (Wuhan, China).

### Peptide binding assay

Peptide binding affinity to HLA-A2 was assessed using T2 cells, a cell line that expresses HLA-A2 molecules but is deficient in antigen presentation. Approximately 50 µg/ml of HLA-A2-specific peptides were co-incubated with 5 × 10^5^ T2 cells at 37°C for 18 h. After incubation, the cells were washed in Phosphate buffered saline (PBS) and further incubated with FITC-conjugated anti-HLA-A2 antibody (BB7.2; BD Biosciences) at 4°C for 45 min. The cells were then washed and re-suspended with 300 µl of PBS and subjected to flow cytometer analysis (FACS Calibur, BD Biosciences). The fluorescence index (FI) was calculated as follows: FI = mean fluorescence intensity (MFI) with the given peptide − MFI without peptide/MFI without peptide. The higher the FI value, the higher the affinity between the peptide and the HLA molecule.

### Peptide/HLA-A2 complex stability assay

T2 cells were collected and placed in 24-well plates (1 × 10^6^/ml), 1 ml in each well. Predictive peptides (50 μM) and β2 microglobulin (2.5 μg/ml) were added, respectively. T2 cells were incubated at 37°C in a 5% CO_2_ sterile incubator for 18 h. After the incubation, T2 cells were washed twice with cold PBS to clear the unbound peptide, then brefeldin A (10 μg/m) was added and incubated for 1 h. Then, cells were washed and incubated at 37°C for 0, 2, 4, and 6 h; FITC-labeled mouse anti-human HLA-A2 monoclonal antibody was added and incubated at 4°C for 30 min under dark conditions. The MFI of cells was detected by flow cytometry after washing twice. The results are expressed as dissociation complex 50 (DC50), meaning the time required for the loss of 50% of the stabilized peptide/complex. Peptides with a high DC50 are more stable when they bind to HLA.

### qRT-PCR

qRT-PCR was performed by the SYBR^®^ Premix Ex Taq™ II kit (Takara, Japan) on the ABI Prism 7900 sequence detection system (Applied Biosystem). The following cycling parameters were used as previously described (13): initial denaturation for 10 min at 95°C; 40 cycles of 15 s at 95°C, and annealing and extension at 60°C for 1 min. Samples were tested in triplicate. The MAGE-D4 primer sequence (29) for qRT-PCR was as follows: 5’-CCAGCTTCTTCTCCTGGATC-3’ (forward) and 5’-GTAACACTGATACCCAAAACATG-3’ (reverse). The HLA-A2 primer sequence (30) for qRT PCR was as follows: 5’-CGTCTAGAATGGCCGTCATGGC-3’ (forward) and 5’-TAGTCGACTCACTTTACAAGCTG-3’ (reverse). MAGE-D4 and HLA-A2 mRNA were determined by the method of 2[−ΔΔCt], which was normalized to GAPDH mRNA.

### Western blot

Cells were digested with trypsin and collected, then lysed in 100 µl of Lysis Buffer (Thermo Fisher Scientific) containing a protease inhibitor mixture. About 75 μg of protein from each sample was separated by 10% SDS-PAGE and transferred to a PVDF membrane. The monoclonal antibodies MAGE-D4 (1:100) and GAPDH (1:1,000) were added, and the secondary antibody was added. An ECL Western blotting substrate (SolarBio) has been developed and imaged in a FluorChem^®^HD2 imaging system. Relative intensities of the bands were normalized with GAPDH and analyzed by ImageJ (NIH, Maryland, USA).

### Pyrosequencing

Genomic DNA was isolated from cells using a DNA Isolation Kit (Vazyme, China). The methylation status of the MAGE-D4 promoter in cells was quantified by the PyroMark Q96 ID pyrosequencing platform (Qiagen), which was performed by Shanghai Geneland Biotech Co., Ltd. The methylation status of each site was analyzed automatically by the accompanying software Pyro Q-CpG (Qiagen). Two regions in the core promoter were detected, which contained 18 CpG sites. Primers used in pyrosequencing were as follows: region 1: 5’-TTGGAGGAAAGGGTTTTTGTTG-3’ (forward) and 5’-CCCCATCCTATCTAAAACTAAATCCTTAC-3’ (reverse); region 2: 5’-GGTTGAGGGGTTTTTGGTGT-3’ (forward) and 5’-AAAAACTCCTATCTAAACCTTAAATC-3’ (reverse). Sequences of the MAGE-D4 promoter and pyrosequencing regions containing CpG sites are listed in **Supplemental Materials**.

### Flow cytometry

To evaluate expression of surface markers, cells were harvested, washed with phosphate-buffered saline, and resuspended in saturating concentrations of monoclonal antibodies or isotype-matched control antibodies overnight at 4°C. The cells were then harvested, washed with PBS buffer three times, and analyzed by a FACScan flow cytometer (BD Biosciences, Franklin Lakes, NJ, USA) using BD Accuri™ C6 Software (BD Biosciences).

### Preparation of peripheral blood mononuclear cells (PBMCs), dendritic cells (DCs), and CD8^+^ T cells

PBMCs were isolated from the peripheral blood samples of 8 HLA-A2^+^ healthy donors, with University Ethics Review Committee-approved, written consent. The effector T lymphocytes and DCs were prepared by our published method (31). CD8^+^ T cells were isolated from PBMCs using CD8^+^ negative selection MicroBeads EasySep™ Human CD8^+^ T Cell Isolation Kit (17953, stemcell) according to the manufacturers.

### T-cell stimulation, CCK-8, and carboxyfluorescein succinimidyl ester (CFSE) lymphocyte proliferation assay

For T-cell stimulation, T cells were cultured with RPMI1640 complete medium containing IL-2 (20 ng/ml) mixed with peptide-pulsed autologous DCs at a ratio of 10:1 and co-cultured for 72 h. The supernatant of DCs and T cells co-cultured for 72h was used to detect IL-2 and γ-IFN. The capacity of peptide-pulsed DCs to stimulate the proliferation of autologous T cells was evaluated using a Cell Counting Kit-8 (CCK-8, Beiltime Biotec, China), as described using our published method (31). CCK-8 solution (20 μl/well) was added in 2 h before the end of culture. Then, the optical density (OD) was measured by microplate tester. The stimulation index (SI) is calculated according to the following formula: SI = (experiment well OD value − background OD value)/(blank control well OD value − background OD value). T-cell proliferation was more obvious in the group with high SI.

CFSE assay was used to further detect the proliferation of T cells stimulated by peptide-pulsed DCs. The cells were suspended at 10^6^/ml with RPMI1640 and added CFSE (5 μM/ml), then incubated at room temperature for 20 min. Fetal bovine serum (FBS, Gbico, USA) was added and incubated for 5 min to terminate the reaction, washed with PBS twice. Then, the peptide-incubated DC was added, incubated for 96 h, and performed flow cytometry analysis.

### Enzyme-linked immunosorbent assay (ELISA)

Production of cytokines in the supernatants was determined using commercially available ELISA kits (Cloud-Clone Corporation, Wuhan, China) according to the protocol of the manufacturer. Approximately 50 μl of supernatant and different concentration standards were added and mixed with 100 μl of horseradish peroxidase (HRP) labeled detection antibody against a 96-well plate, respectively, except for the blank control. After incubation at 37°C for 60 min, we washed the plate five times. Add substrate A and substrate B 50 μl respectively, incubate for 15min in a 37°C incubator in dark. Then, 50 μl of stop solution was added and a 450 nm absorbance value (OD value) was measured within 15 min. The regression equation of the standard curve is calculated according to the concentration of the standard substance and the OD value, and the corresponding sample concentration is calculated on the basis of the regression equation.

### Enzyme-linked Immunospot Assay (ELISPOT)

The number of T cells secreting IFN-γ was measured by ELISPOT using Human IFN-γ precoated ELISPOT kit (Dakewe, Beijing, China). Briefly, RPMI 1640 complete medium was added for 5–10 min to activate the pre-coated plate. T cells (1 × 10^6^/well) stimulated by peptide-pulsed DCs were added to each well and incubated in a 37°C 5% CO2 incubator for 20 h. After lysis in cold water, the biotin-labeled antibody was added and incubated at 37°C for 1 h, then the enzyme-labeled avidin was added and incubated at 37°C for 1 h. The spots present in each well were scanned using the CTL ELISPOT analyzer and counted using the ImmunoSpot Professional Software.

### Assay for cytotoxicity and HLA block

The cytotoxic activity of MAGE-D4 peptide-specific CTLs to glioma cells was determined according to the instructions of the manufacturers by our published method (31). In brief, epigenetic drug-treated target cells (T) U87 and U251 were seeded in 96-well plates and co-cultured with effector cells (E) at indicated dilutions (E:T at 50:1). Four hours later, suspended cells were collected for CD107a flow cytometry, meanwhile, the supernatant was collected and analyzed with granzyme A and perforin ELISA kit (Cloud-Clone Corporation, Wuhan, China) at 450 nm, and the LDH kit (Promega, USA) at 490 nm. The percentage of specific lysis was calculated using the following formula: [(experimental release − spontaneous release)/(maximum release − spontaneous release)] × 100. To determine whether the cytotoxicity depended on the HLA, the HLA-ABC antibody (BD Biosciences, USA) was incubated with target cells at a concentration of 50 μg/ml at 37°C for 40 min. Then, the cytotoxicity assay was measured and calculated as mentioned above.

### *In vivo* xenograft tumor model

Eighteen BALB/C nude female mice aged 4 weeks were provided by the Guangxi Experimental Animal Center. The mice were placed in a light-controlled room at 24 ± 1 °C (light: 7:00–19:00 h, dark: 19:00–7:00 h) and fed under specific pathogen-free conditions. All protocols followed the guidelines of the National Research Council for the Care and Use of Laboratory Animals and were approved by the Animal Ethics Committee of Guangxi Medical University. To develop a xenograft model, 4 × 10^6^ U87 cells were inoculated into the flanks of the mice subcutaneously. Two weeks later, mice simultaneously received intraperitoneal peritumorally injections of TSA (0.5 mg/kg) and 1 × 10^7^ peptides loaded on CD8^+^ T cells, respectively. Every 54 days, tumor volumes were measured simultaneously with a caliper. Mice were sacrificed by euthanasia when visibly ill. Mice injected with negative peptide-induced CTLs and CTLs without peptides were included as controls. The tumor volumes were calculated using the following formula: tumor volume = [(width2 × length)/2].

### Immunohistochemistry (IHC)

Immunochemistry was conducted to detect expressions of CD8 on tissue sections. CD8 antibody (BS-0648R, Bioss, China) was used as the primary antibody. An isotype-matched antibody was used as a control. After incubation overnight with primary antibody at 4°C, sections were subsequently rinsed with PBS and incubated with horseradish peroxidase-labeled IgG (1:500; Thermo Fisher Scientific, USA). The slices were stained with 3,3’-diaminobenzidine according to the instructions of the supplier (Boster, Wuhan, China) and then counterstained with hematoxylin. Then, slides were taken by an Olympus BX53 microscope. Integrated optical density (IOD) was measured by image-Pro Plus software (version 6.0, Media Cybernetics, MD, USA) for images of five randomly selected regions from a slice. The results were expressed as average optical density (AOD = IOD/area).

### Statistical analysis

Data are presented as the means ± standard deviations of at least three independent experiments. SPSS19.0 (SPSS Inc.; Chicago, IL, USA) and GraphPad Prism 5 (San Diego, CA) software were used for statistical analyses and graphical representations. Statistical significance was accepted when P <0.05.

## Results

### High MAGE-D4 expression was correlated with poor prognosis of glioma patients

**Figure 1:**
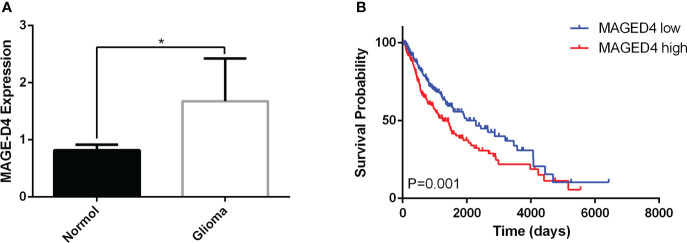
Expression and survival analysis of MAGE-D4 in glioma by GEO and LinkedOmics data. **(A)** Expression of MAGE-D4 in glioma and normal brain tissues. **(B)** Kaplan–Meier survival analysis of 625 glioma patients according to MAGE-D4 expression. *P <0.05.

To understand the expression and prognostic significance of MAGE-D4 mRNA expression, the online data of MAGE-D4 mRNA expression in glioma were collected and analyzed. Analysis of 49 gliomas and four normal brain tissues in the GSE2223 dataset showed that the expression of MAGE-D4 mRNA in glioma was significantly higher than that of normal brain tissue (**Figure 1A**). At the same time, we also analyzed the prognosis of 625 glioma patients in LinkedOmics by a cut-off value based on the median of MAGE-D4 expression. The results demonstrated that the high expression level of MAGE-D4 mRNA in gliomas was also significantly related to the poor prognosis of patients (OS HR = 0.65, 95% CI = 0.50–0.83, logrank P = 0.001). The survival time of patients with high expression of MAGE-D4 mRNA (N = 313) was shorter than that of patients with low expression of MAGE-D4 mRNA (N = 312) (**Figure 1B**). These results suggest that MAGE-D4 was highly expressed in glioma and was associated with a poor prognosis.

### Epigenetic drugs increased expression of MAGE-D4 and HLA-A2 in glioma cells

**Figure 2:**
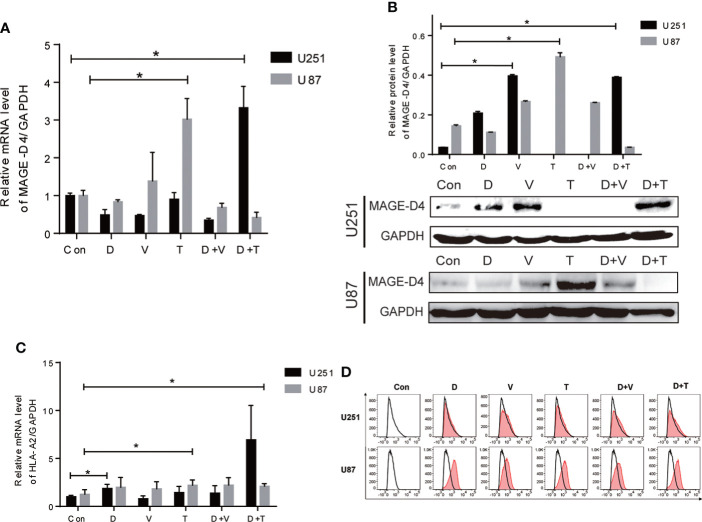
Expression of MAGE-D4 and HLA-A2 in glioma cells treated with epigenectic drugs. **(A)** Relative mRNA expression of MAGE-D4 in U251 and U87 cells treated with epigenectic drugs. **(B)** Relative mRNA expression of HLA-A2 in U251 and U87 cells treated with epigenectic drugs. **(C)** Relative protein expression of MAGE-D4 in U251 and U87 cells treated with epigenetic drugs. **(D)** Expression of HLA-A2 in U251 and U87 cells treated with epigenetic drugs. Con, control; D, DAC; V, VPA; T, TSA; D + V, DAC + VPA; D + T, DAC + TSA; *P <0.05.

As shown in our previous studies, MAGE-D4 expression was correlated with methylation and was elevated by DAC treatment (8, 13), which implied the regulation of MAGE-D4 expression may be involved in the epigenetic regulation. Therefore, in this study, we used epigenetic drugs (DAC, VPA, and TSA alone or in combination) to induce MAGE-D4 expression. As shown in **Figures 2A**, compared to the control, U251 cells expressed higher MAGE-D4 mRNA and protein when treated with DAC+TSA, while U87 cells expressed higher MAGE-D4 mRNA and protein when treated with only TSA.

Since the HLA-A2 molecule is critical for antigen presentation by CD8^+^ T cells, we subsequently examined whether epigenetic drugs may increase HLA-A2 expression in glioma cells. Our results showed that although epigenetic drugs could induce HLA-A2 in both cells, elevated HLA-A2 levels with statistical significance were only exhibited in U251 treated with DAC alone and combined DAC and TSA, and in U87 treated with TSA alone (**Figure 2**). Flow cytometry results also confirmed that epigenetic agents increased HLAA2 antigen expression in gliomas (**Figure 2**).These results imply that gene expression induced by epigenetic drugs is inconsistent in cells.

### MAGE-D4 expression was partly regulated by promoter demethylation

**Figure 3:**
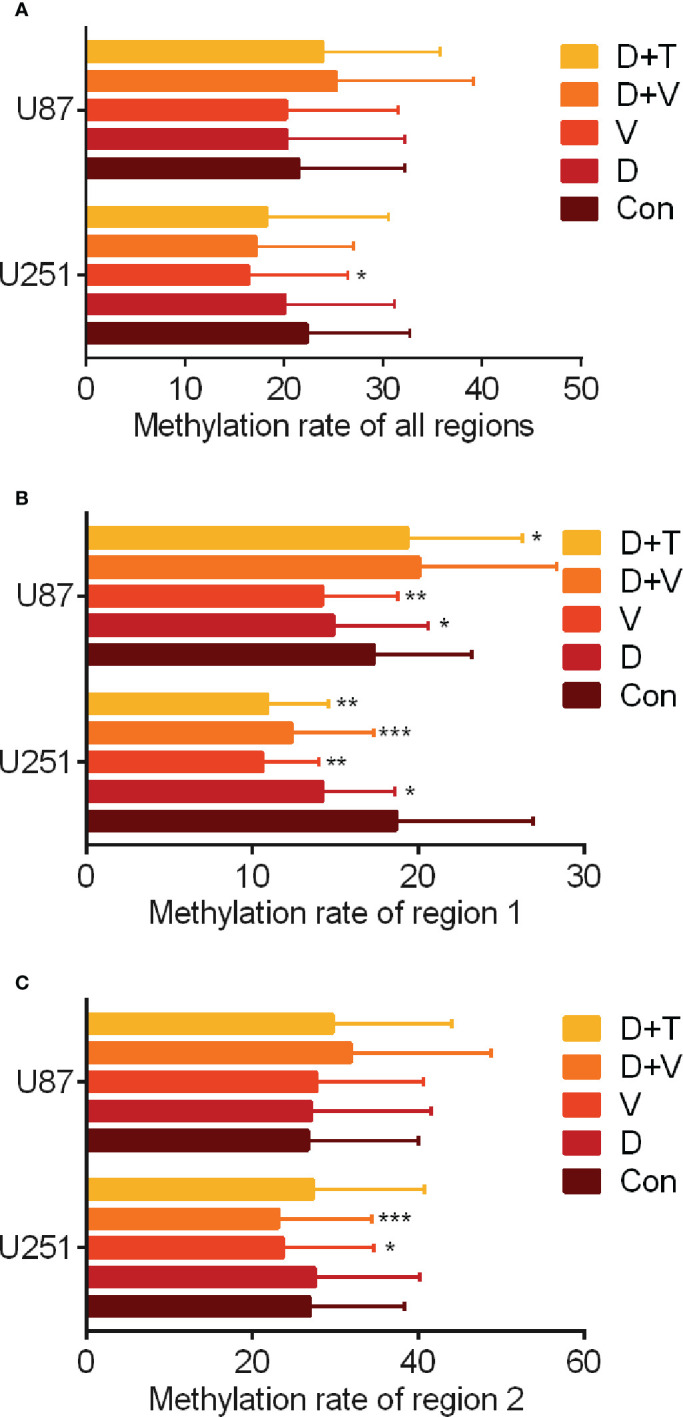
Methylation analysis of MAGE-D4 promoter region in U251 and U87 cells treated with epigenetic drugs. **(A)** Overall level of MAGE-D4 promoter methylation. **(B)** Overall level of MAGE-D4 promoter methylation in region 1. **(C)** Overall level of MAGE-D4 promoter methylation in region 2. Con, control; D, DAC; V, VPA; T, TSA; D + V, DAC + VPA; D + T, DAC + TSA; *P <0.05, **P <0.01, ***P <0.001.

Our previous results showed that epigenetic drugs can enhance the expression of MAGE-D4, but the mechanism is unclear. Since the demethylation of the MAGE-D4 promoter enhances promoter activity (8), pyrosequencing was used to analyze the methylation status of 18 CpG sites in two regions of the MAGE-D4 core promoter region in glioma cells treated with epigenetic drugs. As shown in **Figure 3**, the total methylation levels of regions 1 and 2 in U251 treated by VPA decreased significantly compared with those of the control group. In region 1, the methylation level of U251 cells decreased significantly after treatments with DSA, VPA, DSA + VPA, and DSA + TSA, respectively. And the methylation level of U87 treated with DSA and VPA decreased significantly compared with that of the control group (**Figure 3**). For region 2, the methylation level of U251 decreased significantly in the VPA, DAC + VPA groups, whereas the methylation level of this region in U87 did not significantly decrease (**Figure 3**). These results suggest that methylation levels in the MAGE-D4 core promoter region of glioma cells decreased after epigenetic drug treatment.

### Potential HLA-A*0201 epitopes in MAGE-D4 were screened by prediction programs

To analyze the ability of MAGE-D4 as a targeted therapy for glioma, MAGE-D4 N-terminal antigenic peptides that excluded the MAGE family of homologous peptide were predicted and synthesized. Based on the four prediction programs, IDEB, SYFPEITHI, EPIJEN, and NetMHC, eight native HLA-A2:0201 restricted peptides scored among the top 20 by all the programs were preliminarily selected and named as P1 to P8 (**Table 1**).

**Table 1:** Prediction of HLA-A*0201 restricted epitope peptides of MAGE-D4 N terminal protein by IDEB, SYFPEITHI, EPIJEN and NetMHC.

Peptide	Sequence	Start position	Score							IDEB	SYFPEITHI	EPIJEN	NetMHC
P1	SVQSESYSV	7	216.52	19	8.15	3.7
P2	TLTSFDIHI	47	102.02	18	8.774	3.2
P3	SLGPGLRIL	61	1204.81	30	8.789	5.1
P4	ILSNEPWEL	68	12.41	24	8.134	0.7
P5	ALQLDPETL	88	995.16	24	9.202	4.9
P6	KALAKTRWV	293	3926.6	20	7.810	7.6
P7	VLCLPPRNV	402	3200.87	22	7.939	9.2
P8	CLPPPNVIL	404	819.86	26	8.027	5.0

### MAGE-D4 peptides were selected by affinity binding to HLA-A2 and stability of the peptide/HLA-A2 complex

A peptide binding affinity assay was performed after obtaining potential MAGE-D4 HLA-A*0201 peptides, peptide binding affinity assay was performed. The FI of these peptides reflected binding affinity recorded in T2 cells incubated with each of the eight MAGE-D4 peptides. As shown in **Table 2** (**Figure S1**), P4, P5, P6, P7, and P8 had better affinity than other peptides.

**Table 2:** The HLA-A*0201 binding affinity and stability of peptides.

Peptides	FI	DC50
P1	0.007	>6 h
P2	0.877	>4 h
P3	0.887	>6 h
P4	1.251	>2 h
P5	0.946	>6 h
P6	0.946	>4 h
P7	1.325	<2 h
P8	0.961	>6 h

FI = [mean fluorescence intensity (MFI) of the peptide − MFI background]/[MFI background].

DC50 was defined as the time (h) required for 50% dissociation of the HLA-A*0201/peptide complex stabilized at t = 0 h.

Then, the stability of the peptide/HLA-A2 complex was evaluated by DC50. As shown in **Table 2**, the DC50 of the P1, P2, P3, P5, P6, and P8 complexes was greater than 4 h. Taken together, with combined values of the FI and DC50, P4, P5, and P8 were selected for the further assay.

### MAGE-D4 peptide-pulsed DCs enhanced proliferation and cytokines secretion of T cell

**Figure 4:**
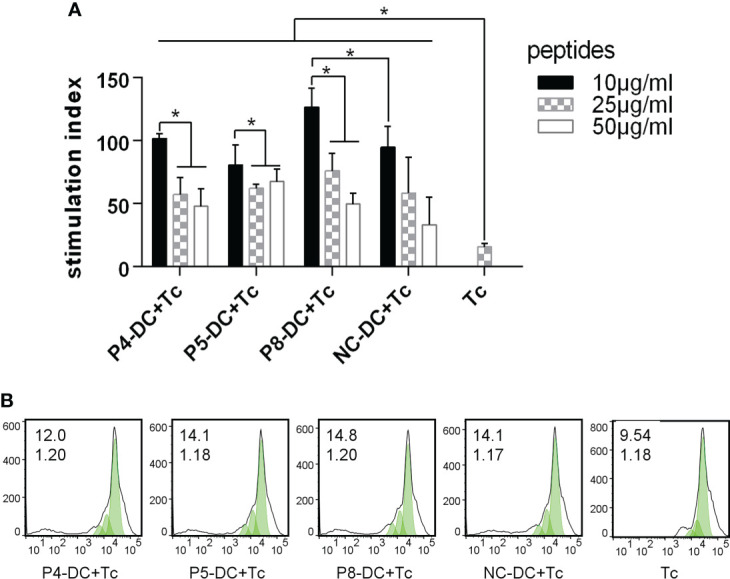
MAGE-D4 peptide-pulsed DCs stimulated the proliferation of T cells. **(A)** CCK8 assay. **(B)** CFSE assay. The value (inset) for the percentage of cells that divided at least once (top left corner) and the proliferation index (bottom left corner) are indicated for each sample. DC, dendritic cell; Tc, T cell; NC, negative control peptide. *P <0.05.

**Figure 5:**
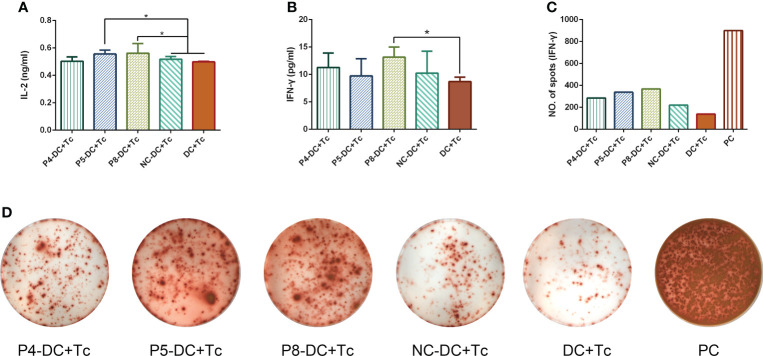
MAGE-D4 peptide pulsed DCs stimulated T cells secreting cytokines. **(A)** ELISA detected IL-2. **(B)** ELISA detected IFN-γ. **(C, D)** ELISOT detected number of T cells secreting IFN-γ. DC, dendritic cell; NC, negative control peptide; Tc,T cell. PC, positive control. *P <0.05.

To determine whether MAGE-D4 peptide-pulsed DCs can activate T cells, we first measured the proliferation of T cells stimulated by the DCs. As shown in **Figure 4**, DCs pulsed with MAGE-D4 peptides P4, P5, and P8 can induce T-cell proliferation compared with DCs without peptide-pulsed. Among these three peptides, the highest SI was demonstrated in T cells stimulated by P8 pulsed DCs. Additionally, considering that DCs pulsed with a peptide concentration of 10 μg/ml had a higher SI for T cells than other concentrations, we chose this concentration for later assays. A CFSE assay was performed to further detect the proliferation of T cells stimulated by peptide-pulsed DCs. As shown in **Figure 4**, the proliferation index and the percentage of divided cells of T cells stimulated by the P4, P5, and P8 pulsed-DCs were higher than T cells stimulated by DCs without peptide-pulsed. Among these three peptides, the T cells stimulated by P8-pulsed DCs scored the highest proliferation index and percentage of divided cells. Then, IL-2 secreted by DC-stimulated T cells was detected by ELISA. The results showed that DCs pulsed by peptides P5 and P8 could stimulate T cells to secrete higher IL-2 than those stimulated by a negative control peptide (**Figure 5**). Simultaneously, we also detect the secretion of IFN-γ by both ELISA and ELISPOT. The result is shown in **Figures 5B**, the peptide P8 pulsed DCs can induce T-cell secreting high levels of IFN-γ compared to T cells alone. With ELISPOT, it was also shown that the number of IFN-γ secreting T cells in the peptide P8 group was more than that in others. Taken together, these results confirmed the ability of DCs pulsed with MAGE-D4 peptides, especially P8, to activate autologous T cells.

### Epigenetic drugs enhanced MAGE-D4-specific cytotoxicity to glioma cells *in vitro*

**Figure 6:**
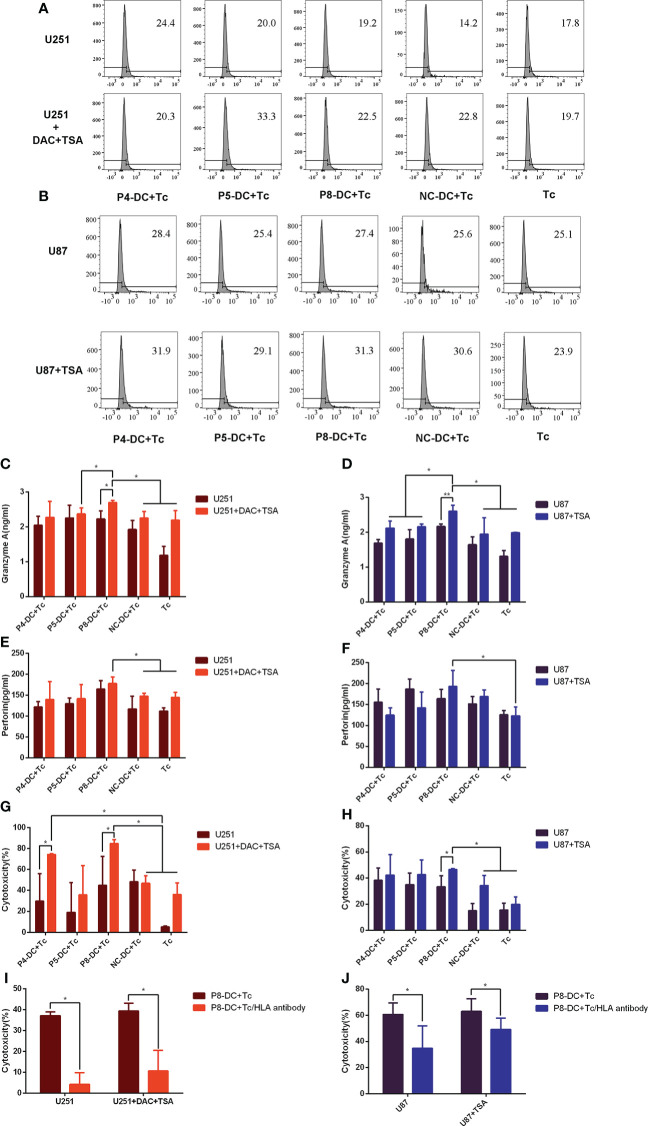
Epigenetic drugs sensitize tumor cells to the cytotoxicity of MAGE-D4-activated T cells. **(A, B)** the ratio of CD107a-expressed T cells stimulated by epigenetic drugs treated U251 **(A)** and U87 **(B)** cells. **(C–F)** the cytokines expression of T cells stimulated by epigenetic drugs treated U251 **(C, E)** and U87 **(D, F)** cells. **(G)** The anti-tumor activity of T cells against U251 treated by DAC and TSA. **(H)** The anti-tumor activity of T cells against U87 treated by TSA. **(I)** The cytotoxicity to DAC + TSA-treated U251 by P8 induced T cells blocked by Anti-HLA antibodies. **(J)** The cytotoxicity to TSA-treated U87 by P8 induced T cells blocked by Anti-HLA antibodies. DC, dendritic cell; Tc, T cell; NC, negative control peptide. *P <0.05.

To determine the cytotoxicity of MAGE-D4-specific T cells, the expression of immunomarkers in CD8^+^ T cells induced by MAGE-D4-specific DC was detected by flow cytometry and ELISA in epigenetic drug-treated glioma cells. Based on the above results that TSA treatment can increase both MAGE-D4 and HLA-A2 expression in U87 cells, and combined treatment of DAC and TSA greatly elevated both MAGE-D4 and HLA-A2 expression in the U251 cells, we first tested the cytotoxicity of MAGE-D4 peptide-specific T cells to DAC + TSA-treated U251 cells and TSA-treated U87 cells. As shown in **Figures 6A, B**, T cells activated by peptide-pulsed DCs expressed more CD107a than untreated T cells, and glioma cells treated with epigenetic drugs induced more CD107a expression. DAC + TSA-treated U251 induced T cells to secrete more granzyme A and perforin, and p8-induced T cells incubated with DAC + TSA-treated U251 secreted the most granzyme A and perforin (**Figures 6C**). TSA-treated U87 induced T cells to secrete more granzyme A and perforin, and P8-induced T cells stimulated by TSA-treated U87 secreted the most granzyme A and perforin (**Figures 6D**). These data suggest that glioma cells treated with epigenetic drugs can enhance the cytotoxicity of MAGE-D4-specific T cells.

A non-radioactive cytotoxicity assay was carried out to investigate whether MAGE-D4 peptide-induced T cells could target the MAGE-D4^+^ HLA-A2^+^ glioma cells. As shown in **Figure 6**, P8-specific T cells had higher cytotoxicity to TSA-treated U87 compared to the controls (T cell alone and NC-T cell groups).It was found that the T cells specific to MAGE-D4 peptides P4 and P8 had cytotoxicity on the DAC + TSA-treated U251 cells with statistical significance (**Figure 6**).

According to the above results, since MAGE-D4 peptides induced T cells had lethality to U87 and U251 cells, we confirm whether the cytotoxicity was HLA dependent. We first applied an anti-HLA antibody to block the HLA on tumor cells, and then the cytotoxicity induced by one of the MAGE-D4 peptides, P8, was assessed. When HLA I was blocked, the killing efficiency was significantly decreased (**Figure 6I**) compared with groups without anti-HLA antibodies. The result implied that MAGE-D4 peptide P8 was presented in association with MHC class I molecules at least in both U87 and U251 cells.

### Tumor growth was inhibited by manipulating MAGE-D4 specific T cells and TSA *in vivo*

**Figure 7:**
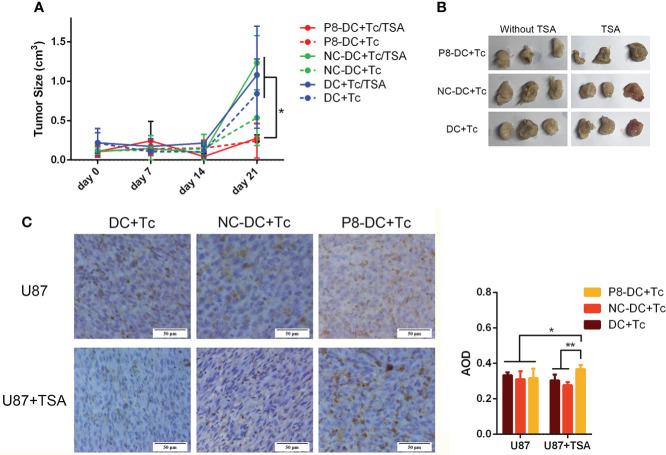
P8-specific T cells inhibited the *in vivo* growth of U87 cell-derived tumors. **(A)** Tumor growth was suppressed by the adoptive transfer of P8-specific T cells. **(B)** Tumors harvested from mice that were inoculated with different groups of T cells and glioma cells. **(C)** CD8^+^ T cells infiltrated glioma tissue *in vivo*. DC, dendritic cell; Tc, T cell; NC, negative control peptide. *P <0.05, **P <0.01, scale bar: 50 μm.

To further verify the cytolytic ability of MAGE-D4-specific T cells *in vivo*, we established a xenotransplantation model. As shown in **Figure 7A**, tumor volume in the P8-specific T cell group was significantly reduced compared with the DC + Tc group and the NC-DC + Tc group, which confirmed the observation *in vitro*. But when the U87 cells were pre-treated with TSA, tumor volume was smaller compared with the U87 group when the two groups of mice were simultaneously inoculated with P8-specific T cells. Tumor growth in the group treated with TSA was slower than that in the group treated without TSA.

CD8^+^ T-cell infiltration *in vivo* tumor tissue was detected by immunohistochemistry. As shown in **Figure 7**, the MAGE-D4-specific T cells infiltrated to the highest degree in TSA-treated U87 glioma tissues *in vivo*. These results suggest that TSA can enhance the invasion of MAGE-D4-specific T cells and decrease tumor growth *in vivo*.

## Discussion

The immune system can recognize and eliminate tumor cells through immunosurveillance, but such anti-tumor responses are usually of low amplitude and inefficient during tumorigenesis. Based on that fact, there are more and more studies on increasing tumor immunotherapy to interrupt tumorigenesis, such as the application of T-cell checkpoint inhibitors (18, 32), DC vaccines (33, 34), epigenetic drugs (17, 34, 35), and so on.

Now MAGE-D4 has been expressed in a range of tumors such as oral squamous cell carcinoma (36, 37), non-small cell lung cancer (38, 39), esophageal squamous cell carcinoma (11, 40), hepatocellular carcinoma (9), renal cell carcinoma (12), colorectal carcinoma (10), and glioma (41), etc. Our previous studies showed that MAGE-D4 was highly expressed in glioma tissues and was immunogenic in glioma patients (13, 42), implying a potential for MAGE-D4 for glioma immunotherapy. However, there is a disadvantage for tumor immunotherapy of MAGE-D4 because of its expression with high heterogeneity in tumors, including gliomas (10, 11, 13). Therefore, it is urgent to eliminate or decrease the heterogeneity in order to develop efficient tumor immunotherapy.

Currently, there are many reports for the use of epigenetic drugs to enhance not only oncogene expression (17, 43, 44) but also HLA expression in hepatoma (45), melanoma (46, 47), Ewing’s sarcoma (48), ovarian cancer (46), leukemia (49), and glioma (35). For instance, given the importance of epigenetic drugs in inducing gene expression and augmenting tumor immunotherapy, some clinical trials have been developed. For instance, a multicenter study for the assessment of combined epigenetic drug histone deacetylase (HDAC) inhibitors to anti-PD1 therapy in the treatment of metastatic uveal melanoma (31, 50). However, as our previous studies showed that MAGE-D4 expression was correlated with methylation and DAC can elevate its expression (8, 13), however, the methylation status of the MAGE-D4 transcription starting point +58 bp to +275 bp region did not change significantly in DAC-treated glioma cells. According to the study by Liu et al. (8), the MAGE-D4 core promoter region was located at promoter −358 bp to +172 bp, so we detected the methylation status of two areas (−111 bp to −32 bp and +29 bp to +112 bp) in the core promoter region in epigenetic drug-treated glioma cells. The results showed that epigenetic agents could demethylate the MAGE-D4 core promoter region CpG. Comprehensive above, it was reasonable to infer the epigenetic regulation in MAGE-D4. It is common sense that CD8^+^ T cells have to recognize both HLA molecules and tumor-specific antigen peptides to kill tumor cells effectively. Therefore, in this study, we preliminarily explored the combination of epigenetic drugs and T cell therapy for glioma treatment.

The most common feature of the MAGE gene family is the existence of a highly conserved MAGE homology domain. This region was located in the position of 421–600 amino acids of MAGE-D4 protein. To explore the specific immune effect of MAGE-D4 protein, we took the 1–420 amino acid region of the N-terminal of MAGE-D4 for peptide prediction. Therefore, eight peptides with the best scores were gained through multiple online platforms, and P4, P5, and P8 were screened out through peptide affinity and peptide/HLA complex stability assays for the next experiment.

Although peptides were used in many recent studies with concentrations ranging from 10–100 μg/ml, such as 20 μg/ml (51), 25 μg/ml (28), and 50 μg/ml (52), it is not always the case that the higher peptide concentration incubated DCs induced the higher activity of T cells. As early as 1964, Mitchison’s immunological study found that high immune doses often failed to induce ideal immune protection effect, which was called “high zone tolerance” (50). In this study, we found that in the range of 10–50 μg/ml, the lower the concentration of MAGE-D4 peptides, the stronger the capacity of DCs to stimulate T-cell proliferation. Therefore, the concentration of peptide-stimulated DC was set at 10 μg/ml. To further screen the peptides with the highest immune activity, CCK8 assay, CFSE assay, ELISA, and ELISPOT were performed, and P8 was found to induce DCs to stimulate the highest T-cell activities of proliferation, IL-2 excretion, and IFN-γ secretion.

A study showed that injection of decitabine-mediated tumor-responsive lymphocytes into peripheral veins of glioblastoma (GBM) patients produced a sustained anti-tumor immune response without treatment-related adverse reactions (53). Tumor treatment with decitabine also resulted in increased levels of MHC and costimulatory molecules (CD80, CD86, and CD40) essential for DC antigen presentation function (54, 55). Our experimental results also showed that MAGE-D4 peptide P8-induced-specific T cells had the highest cytotoxicity rate on glioma cells treated with TSA only or combining TSA and DAC, and its cytotoxicity effect on glioma cells decreased significantly after HLA blocking. *In vivo* experiments also confirmed that these MAGE-D4 peptide P8 induced specific T cells have the strongest inhibitory effect on TSA-treated glioma. These results suggest that TSA combined with MAGE-D4 peptide is a potential therapeutic method for glioma immunotherapy. We speculated that the cytotoxicity effect may be from two hypotheses: firstly, epigenetic drugs inhibited glioma, respectively; Secondly, epigenetic drugs enhanced the killing effect of MAGE-D4 peptide-specific T cells on glioma cells. We prefer the latter hypothesis because our results showed that the killing of glioma cells by specific T cells was HLA and peptide dependent, and epigenetic drugs increased the expression of MAGE-D4 and HLA-A2. Of course, this hypothesis requires further experimental support.

Meanwhile, in our study, with DAC and TSA, the possibility of promoting glioma immunotherapy with combined epigenetic drugs was also explored. Our results showed that the T cells specific to the MAGE-D4 peptide had cytotoxicity in the DAC + TSA-treated U251 cells. To the extent that epigenetic drugs enhance the killing capacity of T cells by increasing the expression of CTA, our results are consistent with those of Ge et al. (17), whose results demonstrated that the administration of epigenetic drugs (DAC + VPA + TSA) could significantly augment the cytotoxicity of ACRBP-specific T cells by increasing the ACRBP expression of tumor cells.

However, to make drugs or tumor-specific T cells circulate from the periphery into the brain to kill gliomas, the difficulty is how to cross the blood–brain barrier (BBB). Notably, brain tumors often disrupt the integrity of the BBB, to a degree that varies from patient tp patient and within the tumor (56). Clinical studies have shown that T cells are transported to active GBM regions after peripheral injection, even though the antitumor activity is limited (57). Further studies have shown that injected intracerebroventricularly (ICV) enables T cells to bypass the BBB and migrate into the brain parenchyma (58). Studies have also focused on intra-arterial drug delivery (59), but technical improvements are needed. Thus, those routes of administration may also be used to bypass BBB.

In conclusion, MAGE-D4 is highly expressed in glioma and correlated with the prognosis of glioma. A novel MAGE-D4 peptide that was identified was capable of inducing MAGE-D4-specific T cells. The application of combined MAGE-D4-specific T cells with epigenetic drugs would enhance cytotoxicity and inhibit growth of glioma *in vitro* and *in vivo*.

## Data availability statement

The original contributions presented in the study are included in the article/**Supplementary Material**. Further inquiries can be directed to the corresponding authors.

## Ethics statement

The studies involving human participants were reviewed and approved by Ethics Committee of Guangxi Medical University. The patients/participants provided their written informed consent to participate in this study. The animal study was reviewed and approved by Animal Ethics Committee of Guangxi Medical University.

## Author contributions

S-QB, Q-M Z, XZ, and CL designed this work, performed experiments. W-XN, HX, FL, and L-NL analyzed data. BL, X, XX, and Y-YG wrote and revised the manuscript. All authors listed have made a substantial, direct, and intellectual contribution to the work and approved it for publication. All authors contributed to the article and approved the submitted version.

## Funding

This work was supported by the National Natural Science Foundation of China (Nos. 81960453, 81860445, and 81460382), the Natural Science Foundation of Guangxi Province (Nos. 2022GXNSFAA035639, 2018GXNSFAA050151, and 2018GXNSFAA281050), the Key Laboratory of Early Prevention and Treatment for Regional High Frequency Tumor (Guangxi Medical University), and the Ministry of Education (Nos. GKE2019-08 and. GKE-ZZ202006).

## Conflict of interest

The authors declare that the research was conducted in the absence of any commercial or financial relationships that could be construed as a potential conflict of interest.

## Publisher’s note

All claims expressed in this article are solely those of the authors and do not necessarily represent those of their affiliated organizations, or those of the publisher, the editors and the reviewers. Any product that may be evaluated in this article, or claim that may be made by its manufacturer, is not guaranteed or endorsed by the publisher.

## References

[1] Siegel RL Miller KD Jemal A. Cancer statistics, 2018: Cancer statistics, 2018. CA Cancer J Clin (2018) 68(1):7–30. doi: 10.3322/caac.21442 29313949

[2] Stupp R Hegi ME Mason WP van den Bent MJ Taphoorn MJB Janzer RC. Effects of radiotherapy with concomitant and adjuvant temozolomide versus radiotherapy alone on survival in glioblastoma in a randomised phase III study: 5-year analysis of the EORTC-NCIC trial. Lancet Oncol (2009) 10(5):459–66. doi: 10.1016/S1470-2045(09)70025-7 19269895

[3] Ghosh S Baker S de Castro DG Kepka L Kumar N Sinaika V. Improved cost-effectiveness of short-course radiotherapy in elderly and/or frail patients with glioblastoma. Radiother Oncol J Eur Soc Ther Radiol Oncol (2018) 127(1):114–20. doi: 10.1016/j.radonc.2018.01.017 29452901

[4] Hasselbalch B Lassen U Hansen S Holmberg M Sørensen M Kosteljanetz M. Cetuximab, bevacizumab, and irinotecan for patients with primary glioblastoma and progression after radiation therapy and temozolomide: a phase II trial. Neuro-Oncol (2010) 12(5):508–16. doi: 10.1093/neuonc/nop063 PMC2940618 20406901

[5] Gil MJ de Las Peñas R Reynés G Balañá C Peréz-Segura P García-Velasco A. Bevacizumab plus irinotecan in recurrent malignant glioma shows high overall survival in a multicenter retrospective pooled series of the Spanish neuro-oncology research group (GEINO). Anticancer Drugs (2012) 23(6):659–65. doi: 10.1097/CAD.0b013e3283534d3e 22634799

[6] Li XF Ren P Shen WZ Jin X Zhang J. The expression, modulation and use of cancer-testis antigens as potential biomarkers for cancer immunotherapy. Am J Transl Res (2020) 12(11):7002–19. PMC7724325 33312347

[7] Shi X Chen X Fang B Ping Y Qin G Yue D. Decitabine enhances tumor recognition by T cells through upregulating the MAGE-A3 expression in esophageal carcinoma. BioMed Pharmacother (2019) 112:108632. doi: 10.1016/j.biopha.2019.108632 30797153

[8] Liu C Ge Y Luo B Xie X Shen N Nong W. Synergistic regulation of methylation and SP1 on MAGE-D4 transcription in glioma. Am J Transl Res (2021) 13(4):2241–55. PMC8129322 34017386

[9] Takami H Kanda M Oya H Hibino S Sugimoto H Suenaga M. Evaluation of MAGE-D4 expression in hepatocellular carcinoma in Japanese patients. J Surg Oncol (2013) 108(8):557–62. doi: 10.1002/jso.23440 24068544

[10] Zhang QM He SJ Shen N Luo B Fan R Fu J. Overexpression of MAGE-D4 in colorectal cancer is a potentially prognostic biomarker and immunotherapy target. Int J Clin Exp Pathol (2014) 7(7):3918–27. PMC4129003 25120768

[11] Uno Y Kanda M Sato Y Shimizu D Umeda S Hattori N. Expression, function, and prognostic value of MAGE-D4 protein in esophageal squamous cell carcinoma. Anticancer Res (2019) 39(11):6015–23. doi: 10.21873/anticanres.13807 31704827

[12] Kramer BF Schoor O Kruger T Reichle C Muller M Weinschenk T. MAGED4-expression in renal cell carcinoma and identification of an HLA-A*25-restricted MHC class I ligand from solid tumor tissue. Cancer Biol Ther (2005) 4(9):943–8. doi: 10.4161/cbt.4.9.1907 16082191

[13] Zhang QM Shen N Xie S Bi SQ Luo B Lin YD. MAGED4 expression in glioma and upregulation in glioma cell lines with 5-Aza-2’-Deoxycytidine treatment. Asian Pac J Cancer Prev (2014) 15(8):3495–501. doi: 10.7314/APJCP.2014.15.8.3495 24870746

[14] Yan J Wen J Wei ZD Li XS Li P Xiao SW. Prognostic and clinicopathological value of melanoma-associated antigen D4 in patients with glioma. Oncol Lett (2018) 15(4):4151–60. doi: 10.3892/ol.2018.7884 PMC5835852 29541180

[15] Schäfer P Paraschiakos T Windhorst S. Oncogenic activity and cellular functionality of melanoma associated antigen A3. Biochem Pharmacol (2021) 192:114700. doi: 10.1016/j.bcp.2021.114700 34303709

[16] Neumann F Kaddu-Mulindwa D Widmann T Preuss KD Held G Zwick C. EBV-transformed lymphoblastoid cell lines as vaccines against cancer testis antigen-positive tumors. Cancer Immunol Immunother CII (2013) 62(7):1211–22. doi: 10.1007/s00262-013-1412-z PMC11028802 23619976

[17] Ge YY Zhang QM Liu C Zeng X Nong WX Chen F. Combined treatment with epigenetic agents enhances anti-tumor activity of T cells by upregulating the ACRBP expression in hepatocellular carcinoma. Am J Transl Res (2021) 13(7):7591–609. PMC8340224 34377237

[18] Wu Y Sang M Liu F Zhang J Li W Li Z. Epigenetic modulation combined with PD-1/PD-L1 blockade enhances immunotherapy based on MAGE-A11 antigen-specific CD8+T cells against esophageal carcinoma. Carcinogenesis (2020) 41(7):894–903. doi: 10.1093/carcin/bgaa057 32529260

[19] Kim VM Pan X Soares KC Azad NS Ahuja N Gamper CJ. Neoantigen-based EpiGVAX vaccine initiates antitumor immunity in colorectal cancer. JCI Insight (2020) 5(9):136368. doi: 10.1172/jci.insight.136368 32376802 PMC7253020

[20] Bensaid D Blondy T Deshayes S Dehame V Bertrand P Grégoire M. Assessment of new HDAC inhibitors for immunotherapy of malignant pleural mesothelioma. Clin Epigenetics (2018) 10:79. doi: 10.1186/s13148-018-0517-9 29946373 PMC6006850

[21] Li XF Zhang X Chen Y Zhang KL Liu XJ Li JP. An analysis of HLA-a, -b, and -DRB1 allele and haplotype frequencies of 21,918 residents living in liaoning, China. PloS One (2014) 9(4):e93082. doi: 10.1371/journal.pone.0093082 24691290 PMC3972227

[22] Vasaikar SV Straub P Wang J Zhang B. LinkedOmics: analyzing multi-omics data within and across 32 cancer types. Nucleic Acids Res (2018) 46(D1):D956–63. doi: 10.1093/nar/gkx1090 PMC5753188 29136207

[23] Bredel M Scholtens DM Harsh GR Bredel C Chandler JP Renfrow JJ. A network model of a cooperative genetic landscape in brain tumors. JAMA (2009) 302(3):261–75. doi: 10.1001/jama.2009.997 PMC4447713 19602686

[24] Fleri W Paul S Dhanda SK Mahajan S Xu X Peters B. The immune epitope database and analysis resource in epitope discovery and synthetic vaccine design. Front Immunol (2017) 8:278. doi: 10.3389/fimmu.2017.00278 28352270 PMC5348633

[25] Andreatta M Nielsen M. Gapped sequence alignment using artificial neural networks: application to the MHC class I system. Bioinforma Oxf Engl (2016) 32(4):511–7. doi: 10.1093/bioinformatics/btv639 PMC6402319 26515819

[26] Doytchinova IA Guan P Flower DR. EpiJen: a server for multistep T cell epitope prediction. BMC Bioinf (2006) 7:131. doi: 10.1186/1471-2105-7-131 PMC1421443 16533401

[27] Rammensee H Bachmann J Emmerich NP Bachor OA Stevanović S. SYFPEITHI: database for MHC ligands and peptide motifs. Immunogenetics (1999) 50(3–4):213–9. doi: 10.1007/s002510050595 10602881

[28] Lim KP Chun NAL Gan CP Teo SH Rahman ZAA Abraham MT. Identification of immunogenic MAGED4B peptides for vaccine development in oral cancer immunotherapy. Hum Vaccines Immunother (2014) 10(11):3214–23. doi: 10.4161/hv.29226 PMC4517457 25483651

[29] Johnson JM Castle J Garrett-Engele P Kan Z Loerch PM Armour CD. Genome-wide survey of human alternative pre-mRNA splicing with exon junction microarrays. Science (2003) 302(5653):2141–4. doi: 10.1126/science.1090100 14684825

[30] Gilbert W. Recombinant DNA research: government regulation. Science (1977) 197(4300):208. doi: 10.1126/science.11643384 11643384

[31] Luo B Yun X Li J Fan R Guo WW Liu C. Cancer-testis antigen OY-TES-1 expression and immunogenicity in hepatocellular carcinoma. Curr Med Sci (2020) 40(4):719–28. doi: 10.1007/s11596-020-2241-x 32862383

[32] Jespersen H Olofsson Bagge R Ullenhag G Carneiro A Helgadottir H Ljuslinder I. Concomitant use of pembrolizumab and entinostat in adult patients with metastatic uveal melanoma (PEMDAC study): protocol for a multicenter phase II open label study. BMC Cancer (2019) 19(1):415. doi: 10.1186/s12885-019-5623-3 31046743 PMC6498539

[33] Sun L Kong B Sheng X Sheu JJC Shih IM. Dendritic cells transduced with rsf-1/HBXAP gene generate specific cytotoxic T lymphocytes against ovarian cancer *in vitro* . Biochem Biophys Res Commun (2010) 394(3):633–8. doi: 10.1016/j.bbrc.2010.03.038 20226169

[34] Lee DH. Dendritic cells-based vaccine and immune monitoring for hepatocellular carcinoma. Korean J Physiol Pharmacol Off J Korean Physiol Soc Korean Soc Pharmacol (2010) 14(1):11–4. doi: 10.4196/kjpp.2010.14.1.11 PMC2835977 20221274

[35] Natsume A Wakabayashi T Tsujimura K Shimato S Ito M Kuzushima K. The DNA demethylating agent 5-aza-2’-deoxycytidine activates NY-ESO-1 antigenicity in orthotopic human glioma. Int J Cancer (2008) 122(11):2542–53. doi: 10.1002/ijc.23407 18240144

[36] Cheong SC Chandramouli GVR Saleh A Zain RB Lau SH Sivakumaren S. Gene expression in human oral squamous cell carcinoma is influenced by risk factor exposure. Oral Oncol (2009) 45(8):712–9. doi: 10.1016/j.oraloncology.2008.11.002 19147396

[37] Chong CE Lim KP Gan CP Marsh CA Zain RB Abraham MT. Over-expression of MAGED4B increases cell migration and growth in oral squamous cell carcinoma and is associated with poor disease outcome. Cancer Lett (2012) 321(1):18–26. doi: 10.1016/j.canlet.2012.03.025 22459352

[38] Ma QY Pang LW Chen ZM Zhu YJ Chen G Chen J. The significance of MAGED4 expression in non-small cell lung cancer as analyzed by real-time fluorescence quantitative PCR. Oncol Lett (2012) 4(4):733–8. doi: 10.3892/ol.2012.786 PMC3506701 23205092

[39] Ito S Kawano Y Katakura H Takenaka K Adachi M Sasaki M. Expression of MAGE-D4, a novel MAGE family antigen, is correlated with tumor-cell proliferation of non-small cell lung cancer. Lung Cancer (2006) 51(1):79–88. doi: 10.1016/j.lungcan.2005.08.012 16225959

[40] Oya H Kanda M Takami H Hibino S Shimizu D Niwa Y. Overexpression of melanoma-associated antigen D4 is an independent prognostic factor in squamous cell carcinoma of the esophagus. Esophagus (2015) 28(2):188–95. doi: 10.1111/dote.12156 24147998

[41] Arora M Kumari S Singh J Chopra A Chauhan SS. Downregulation of brain enriched type 2 MAGEs is associated with immune infiltration and poor prognosis in glioma. Front Oncol (2020) 10:573378. doi: 10.3389/fonc.2020.573378 33425727 PMC7787151

[42] He SJ Gu YY Yu L Luo B Fan R Lin WZ. High expression and frequently humoral immune response of melanoma-associated antigen D4 in glioma. Int J Clin Exp Pathol (2014) 7(5):2350–60. PMC4069931 24966945

[43] Souri Z Jochemsen AG Versluis M Wierenga APA Nemati F van der Velden PA. HDAC inhibition increases HLA class I expression in uveal melanoma. Cancers (2020) 12(12):E3690. doi: 10.3390/cancers12123690 33316946 PMC7763827

[44] Serrano A Tanzarella S Lionello I Mendez R Traversari C Ruiz-Cabello F. Rexpression of HLA class I antigens and restoration of antigen-specific CTL response in melanoma cells following 5-aza-2’-deoxycytidine treatment. Int J Cancer (2001) 94(2):243–51. doi: 10.1002/ijc.1452 11668505

[45] Xiao WH Sanren GW Zhu JH Li QW Kang HR Wang RL. Effect of 5-aza-2’-deoxycytidine on immune-associated proteins in exosomes from hepatoma. World J Gastroenterol (2010) 16(19):2371–7. doi: 10.3748/wjg.v16.i19.2371 PMC2874141 20480522

[46] Adair SJ Hogan KT. Treatment of ovarian cancer cell lines with 5-aza-2’-deoxycytidine upregulates the expression of cancer-testis antigens and class I major histocompatibility complex-encoded molecules. Cancer Immunol Immunother CII (2009) 58(4):589–601. doi: 10.1007/s00262-008-0582-6 18791715 PMC11029901

[47] Ny L Jespersen H Karlsson J Alsén S Filges S All-Eriksson C. The PEMDAC phase 2 study of pembrolizumab and entinostat in patients with metastatic uveal melanoma. Nat Commun (2021) 12(1):5155. doi: 10.1038/s41467-021-25332-w 34453044 PMC8397717

[48] Krishnadas DK Bao L Bai F Chencheri SC Lucas K. Decitabine facilitates immune recognition of sarcoma cells by upregulating CT antigens, MHC molecules, and ICAM-1. Tumour Biol J Int Soc Oncodevelopmental Biol Med (2014) 35(6):5753–62. doi: 10.1007/s13277-014-1764-9 24584817

[49] Poláková K Bandzuchová E Kuba D Russ G. Demethylating agent 5-aza-2’-deoxycytidine activates HLA-G expression in human leukemia cell lines. Leuk Res (2009) 33(4):518–24. doi: 10.1016/j.leukres.2008.08.003 18823661

[50] N A MITCHISON Induction of immunological paralysis in two zones of dosage. Proc R Soc Lond B Biol Sci (1964) 161(983):275–92. doi: 10.1098/rspb.1964.0093 14224412

[51] Tu X Li S Zhao L Xiao R Wang X Zhu F. Human leukemia antigen-A*0201-restricted epitopes of human endogenous retrovirus W family envelope (HERV-W env) induce strong cytotoxic T lymphocyte responses. Virol Sin (2017) 32(4):280–9. doi: 10.1007/s12250-017-3984-9 PMC6598903 28840564

[52] Chai SJ Fong SCY Gan CP Pua KC Lim PVH Lau SH. *In vitro* evaluation of dual-antigenic PV1 peptide vaccine in head and neck cancer patients. Hum Vaccines Immunother (2019) 15(1):167–78. doi: 10.1080/21645515.2018.1520584 PMC6363157 30193086

[53] Kirkin AF Dzhandzhugazyan KN Guldberg P Fang JJ Andersen RS Dahl C. Adoptive cancer immunotherapy using DNA-demethylated T helper cells as antigen-presenting cells. Nat Commun (2018) 9(1):1-12. doi: 10.1038/s41467-018-03217-9 PMC5840134 29511178

[54] Chen X Pan X Zhang W Guo H Cheng S He Q. Epigenetic strategies synergize with PD-L1/PD-1 targeted cancer immunotherapies to enhance antitumor responses. Acta Pharm Sin B (2020) 10(5):723–33. doi: 10.1016/j.apsb.2019.09.006 PMC7276686 32528824

[55] Dubovsky JA McNeel DG Powers JJ Gordon J Sotomayor EM Pinilla-Ibarz JA. Treatment of chronic lymphocytic leukemia with a hypomethylating agent induces expression of NXF2, an immunogenic cancer testis antigen. Clin Cancer Res Off J Am Assoc Cancer Res (2009) 15(10):3406–15. doi: 10.1158/1078-0432.CCR-08-2099 19401350

[56] Sarkaria JN Hu LS Parney IF Pafundi DH Brinkmann DH Laack NN. Is the blood-brain barrier really disrupted in all glioblastomas? a critical assessment of existing clinical data. Neuro-Oncol (2018) 20(2):184–91. doi: 10.1093/neuonc/nox175 PMC5777482 29016900

[57] O’Rourke DM Nasrallah MP Desai A Melenhorst JJ Mansfield K Morrissette JJD. A single dose of peripherally infused EGFRvIII-directed CAR T cells mediates antigen loss and induces adaptive resistance in patients with recurrent glioblastoma. Sci Transl Med (2017) 9(399):eaaa0984. doi: 10.1126/scitranslmed.aaa0984 28724573 PMC5762203

[58] Fisher Y Strominger I Biton S Nemirovsky A Baron R Monsonego A. Th1 polarization of T cells injected into the cerebrospinal fluid induces brain immunosurveillance. J Immunol Baltim Md 1950 (2014) 192(1):92–102. doi: 10.4049/jimmunol.1301707 24307730

[59] Rechberger JS Thiele F Daniels DJ. Status quo and trends of intra-arterial therapy for brain tumors: A bibliometric and clinical trials analysis. Pharmaceutics (2021) 13(11):1885. doi: 10.3390/pharmaceutics13111885 34834300 PMC8625566

