# Supplementary material for: Combined treatment with epigenetic agents enhances anti-tumor activity of MAGE-D4 peptide-specific T cells by upregulating the MAGE-D4 expression in glioma
Source: Front Oncol. 2022 Aug 3;12:873639. doi: 10.3389/fonc.2022.873639 (PMC9382192; doi:10.3389/fonc.2022.873639)
Supplement: Supplementary file 1 [file DataSheet_1.docx]

## 1. Sequences of MAGE-D4 promoter and pyrosequencing regions

GTATGTCTGGGGGCCAGAGAGTTGGCTAGTGGAGAAGCACACAAAGCGAAGTGCCATCCTCTGGCCATGTCCATTTCGTAGCCCCGCAGGTTGAGGATTTCCACTTGTTGCAGTTAGAGACCCAGCTTATTAATTGTGAGACCTCACTAATTGTGACCTAAGGGGTCTTGCCGGGGAAACGGGGCATAGTGTATCCCAGACCAGGCTGGAGGAGTTTGGGTGAAAGGGCAGGGCAGGACAGAGAAGCATTGTGCAAAAGTGGGGGGACTGAGAAGAGATGATTGGGTGGGTCGAGCTGCACTGCAGGGTATGAGTGTGCGCGCCCGCGGTAGGAGGAGGAGGAGGAGGAGAGGTGAAGTGGATCTGGCGGGAAGAGCCCCCCGCACCCTACCTTCGACCCCACCGCCCACCCACCCGCGGAATCGCATGCGCACTGGAGACCTGGAGGAAAGGGCTTTTGTTGGGAAAGCGGGCGGGCTGGAGGGGTCCGCGCATGCGCAGGCTACCCAGCCGCGGGGGGTGCACGGAGAAAAGGGGCGGGGTGGTCCGGGCTGCTGTGCTGGCAGCAGTAGGCGAGGGCGCGGCTGCGGGGTTCCTGGTGCTGAGGACGGACGCCATTGGAGTTCCCGAGAAGGTAAGGATCCAGCCCCAGACAGGACCGGGAGAGGGCGAGTGGAACCCGACACGCTGCGCCCTCCCTCCGCCTCCGGATCTGAACAAAGCCCAAGCACTCAGAACCGGAACCCCATTAGACCCAAGGTCTAGATAGGAGCCCCCATCACCATCAGACCCAGGCGCCCCGATCTGAGCCCTACTGAAACCGGAGCCCAGGATCCTCACCCCTTTAGCAGACCCGTGTGCTCCGAGCTGAGCTCCCTTGGACCTGAGGCCCCACCCCCACCCCAACCACTCCTAGATTACTCGAACCGAGCTGACCGCTTGCCCCCTTCCTGGAGTGCCCAGTCCTCGCGTTTGAGATCTGCAGCGCTCCGATTGGAGCCTCACCTAGGTCTGAGGCCCCCACTCCATCCGCCTCTAGTGCTCGAGTCTGAGCCCCACCTAGGCCCCCCGCCCGGACCTAGCCAAAGGTCCCTGGGGTTCTGTTTCGCAGAGCTTGCGGCTTGCCACTGTCCCTGTTGTCTGAGCTCTCCCATCTGCTCCCCCTTCATCCCGGTCCCCTTCTCTGGCCCGTAAATCCAAACCCTTTGTTTCTCTCTTCCCCAATGCATTCCCTTTGGGACTCTTCGGACCCCAGCCCTCCAGAACACCCCCTCGTCAAATCTAGCCGCTGGGATGGCGAGCCTGCCCATCCTAAACTCCGCTTTCAGTGCGGCGCCTCCTGCGACCTCC

Underline: core promoter region; Yellow highlight: Region 1; Blue highlight: Region 2; Red CG: detectable CpG sites; CG in the frame: CpG site detected by pyrosequencing.

## 2. Supplemental figure legend

**Figure S1.** The HLA-A*0201 binding affinity and stability of peptides. FI = [mean fluorescence intensity (MFI) of the peptide - MFI background]/[MFI background]
